# Anemia Control in Kidney Transplant Recipients Using Once-Monthly Continuous Erythropoietin Receptor Activator: A Prospective, Observational Study

**DOI:** 10.1155/2014/179705

**Published:** 2014-05-04

**Authors:** Klemens Budde, Thomas Rath, Volker Kliem

**Affiliations:** ^1^Department of Nephrology, Charité, Medical University, 10117 Berlin, Germany; ^2^Department of Nephrology and Transplantation Medicine, Westpfalz Hospital, 67655 Kaiserslautern, Germany; ^3^Nephrology Center of Lower Saxony and Transplant Center, Vogelsang 105, 34346 Hann. Münden, Germany

## Abstract

In a multicenter, prospective, observational study of 279 kidney transplant recipients with anemia, the efficacy and safety of once-monthly continuous erythropoietin receptor activator (C.E.R.A.) were assessed to a maximum of 15 months. The main efficacy variable was the proportion of patients achieving a hemoglobin level of 11-12 g/dL at each of visits between months 7 and 9. At study entry, 224 patients (80.3%) were receiving erythropoiesis stimulating agent (ESA) therapy including darbepoetin alfa (98), epoetin beta (61), and C.E.R.A. (45). The mean (SD) time between C.E.R.A. applications was 34.0 (11.9) days. Among 193 patients for whom efficacy data were available, mean (SD) hemoglobin was 11.1 (0.99) g/dL at study entry, 11.5 (1.1) g/dL at month 7, 11.6 (1.3) g/dL at month 9, and 11.4 (1.1) g/dL at month 15. During months 7–9, 20.7% of patients had all hemoglobin values within the range 11-12 g/dL and 64.8% were within 10–13 g/dL. Seven patients (2.5%) discontinued C.E.R.A. due to adverse events or serious adverse events. In this observational trial under real-life conditions, once-monthly C.E.R.A. therapy achieved stable hemoglobin levels in stable kidney transplant recipients with good tolerability, and with no requirement for any dose change in 43% of patients.

## 1. Introduction

Anemia is virtually universal at the time of kidney transplantation [1]. Chronic kidney disease (CKD) blunts erythropoietin production [2], a proanemic effect that is compounded by other factors such as accelerated erythrocyte destruction and widespread use of concomitant medication such as ACE inhibitors and ARBs [3]. Following transplantation, the prevalence of anemia declines sharply as renal function is restored but low hemoglobin (Hb) levels persist in a worryingly large proportion of cases due to multiple factors such as suboptimal renal function, cardiovascular medication, and certain immunosuppressive therapies [4, 5]. In the largest series to date, an analysis of 5,834 kidney transplant recipients at 10 European outpatient transplant clinics detected anemia in 42% of patients based on the American Society of Transplantation anemia guidelines (Hb ≤ 13.0 g/dL in males and ≤ 12.0 g/dL in females) [6]. Using the same thresholds, large single-center cohort studies have found that 30–35% of kidney transplant patients have anemia [7–9]. In nontransplant CKD populations, anemia is predictive of cardiovascular events [10], mortality [11, 12], and diminished quality of life [13]. Posttransplant anemia is significantly associated with increased death-censored [14, 15] and all-cause [9, 16, 17] graft loss, probably cardiovascular events [18] and possibly mortality [16–19], although causative relationships are not certain and anemia may be a marker for other pathologic processes.

Posttransplant anemia remains undertreated. In 2003, the Transplant European Survey on Anemia Management (TRESAM) analyzed a cohort of 4,263 patients from 72 centers in 17 countries and found that only 18% of patients with Hb < 11 g/dL were receiving erythropoiesis stimulating agents (ESAs) [20]. In a follow-up study, five years later, this proportion had increased to just 24% [6]. This low intervention rate may partly reflect missed diagnoses and safety concerns about ESA therapy to target high Hb levels [21–24], but also the relative paucity of robust trials of ESA therapy in kidney transplantation. Findings from nontransplant populations cannot necessarily be extrapolated to kidney transplantation since the mechanisms underlying anemia and epoetin resistance may differ [3]. The available evidence in transplant patients, however, indicates that ESA therapy is effective in increasing Hb levels, based on data from two small randomized trials undertaken in the early posttransplant period [25, 26], a nonrandomized prospective multicenter study [27], an observational trial [28], and retrospective analyses [29, 30]. Correction of posttransplant anemia with ESA is associated with improved quality of life [27]. However, questions remain. Results from the CHOIR [23] and CREATE [24] studies raised doubts about Hb targets in patients with CKD, leading to revised recommendations [31], an issue that is largely unexplored in kidney transplantation. Moreover, interventional studies typically report mean Hb values, and data relating to Hb fluctuation in individual kidney transplant patients are lacking.

Studies of ESA therapy in kidney transplantation have generally used epoetin or darbepoetin. Using these products, dosing is typically required thrice weekly for epoetin alfa or epoetin beta in the maintenance phase and at least every 2–4 weeks for darbepoetin. Continuous erythropoietin receptor activator (C.E.R.A.) is a modified recombinant human erythropoietin which has been designed to have a longer half-life than other ESA preparations [32]. As a result, correction of anemia can be achieved with dosing every two weeks in hemodialysis patients and once a month in nondialysis CKD patients, while during the maintenance phase, all patients require only once-monthly dosing [33], offering greater convenience for patients and healthcare staff.

The current multicenter, prospective, observational study was designed to evaluate the efficacy and safety of C.E.R.A. in anemic kidney transplant recipients, either administered de novo or following conversion from more frequently administered ESA therapies. The study design was developed with several points in mind. First, results from the CHOIR [23] and CREATE [24] studies raised doubts about Hb targets in patients with CKD, leading to revised recommendations [33]. However, Hb levels in routine practice are largely undocumented in kidney transplantation. Second, recent Phase III trials of C.E.R.A. targeted an Hb level of not more than 13 g/dL [34, 35], but the extent to which this upper threshold is maintained in kidney transplant patients during routine management was unknown. Lastly, interventional studies typically report mean Hb values, and data relating to Hb fluctuation in individual kidney transplant patients are lacking.

## 2. Methods

### 2.1. Study Design and Conduct

This was a prospective, noninterventional, single-arm study of kidney transplant patients receiving C.E.R.A. therapy at 37 German transplant centers, which took place during the period from September 2007 to November 2011. The initial observation period of nine months was extended to 15 months, as permitted in the study protocol, in order to gather longer-term data, especially with regard to the phenomenon of Hb cycling.

The study was undertaken in accordance with the principles laid down in the Declaration of Helsinki and Good Clinical Practice. The study protocol was approved by the ethics committee at the Medizinische Hochschule Hannover, Hannover, Germany. All participants provided written informed consent.

### 2.2. Patient Population

Patients were eligible for inclusion if they had received a kidney transplant at least three months prior to study entry and had stable graft function (defined as ≤25% loss of function in the previous three months) and their physicians had decided to administer C.E.R.A. therapy prior to study entry. All patients were required to have a life expectancy of at least nine months (the initial planned duration of the study period), with no active malignant disease or acute infection and no acute blood loss or decrease in Hb level, in the four weeks prior to inclusion. Patients on dialysis were excluded. Patients were to be withdrawn from the study if they required dialysis at any point or if an ESA other than C.E.R.A. was initiated.

### 2.3. Medication

Prior to study entry, any ESA therapy was administered by the physician according to local practice and the summary of product characteristics of the selected ESA. All patients received C.E.R.A. therapy from study entry, prescribed according to local practice.

### 2.4. Evaluation

Study visits were scheduled to take place at study entry and once a month throughout the 15-month observation period, with a minimum of fifteen postbaseline visits. For patients enrolled prior to extension of the study to 15 months, a minimum of nine postbaseline visits were required. At study entry, the following data were collected: demographics, type of transplant, time since transplantation, duration and regimen of previous ESA therapy, baseline Hb concentration prior to C.E.R.A. administration, additional laboratory values (iron status, blood count, liver function, estimated glomerular filtration rate (eGFR), C-reactive protein (CRP) and vitamin B_12_ concentrations), and concomitant disease/medication. Subsequent study visits included recording of Hb value prior to C.E.R.A. administration, collection of additional laboratory data, and changes in concomitant disease/medication. GFR was estimated using the abbreviated four-variable Modification of Diet in Renal Disease (MDRD [36]) formula. Adverse events were documented, including duration, severity, whether the event was regarded as serious, and causal relationship with C.E.R.A. Serious adverse events were defined as those which were life-threatening or fatal, required unplanned hospitalization or prolonged hospitalization, resulted in persistent or significant disability or incapacity, or were regarded as an important medical event. Additionally, a decrease in Hb concentration of >2 g/dL, any occurrence of pure red cell aplasia or production of anti-epoetin antibodies was to be handled as serious adverse drug reactions of special interest.

Data were recorded by study investigators on printed forms and checked at the participating center for completeness, and then entered independently to a database by an independent research organization (M.A.R.C.O. GmbH & Co KG, 40227 Düsseldorf, Germany) which was also responsible for clarifying discrepancies on the submitted forms.

### 2.5. Statistical Analysis

The main efficacy variable was the proportion of patients (“responders”) achieving an Hb concentration of 11-12 g/dL at each of visits 7, 8, and 9, that is, after a 7–9 month period for C.E.R.A. dose titration. Following extension of the study to a 15-month observation period, the proportion of patients within each of these two Hb ranges was also calculated for the periods covering months 7 to 12 and months 7 to 15. In additional prespecified analyses, the proportion of patients within the Hb ranges 10–12, 10–13 g/dL and 11–13 g/dL were also calculated for each of these time periods.

The sample size calculation showed that a total of 300 patients were required, based on a maximum responder rate of 85%, a mean accuracy (mean confidence interval width) of 5%, a drop-out rate of approximately 30%, and a significance level of 5%. All analyses are presented descriptively. Confidence intervals are reported where appropriate. Efficacy analyses were performed in the efficacy population, defined as all patients who provided at least one measurement of Hb concentration and received at least one dose of C.E.R.A. during months 7–9 of the study, did not receive any ESA therapy other than C.E.R.A. during the study, met the inclusion/exclusion criteria as confirmed in writing by the investigator, and did not have other major protocol violations. Safety analyses were performed on all patients who received at least one dose of C.E.R.A. For patients in whom C.E.R.A. therapy was terminated before the end of the observation period, data were analyzed to the point of discontinuation.

## 3. Results

### 3.1. Patient Population

**Figure 1:**
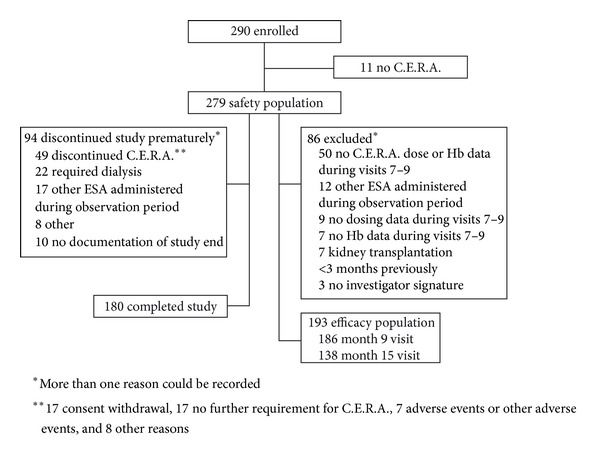
Patient disposition.

In total, 290 patients were enrolled to the study. Of these, 11 did not receive C.E.R.A. such that the safety population comprised 279 patients. The efficacy population included 193 patients, with exclusion most frequently due to absence of C.E.R.A. dosing and/or a missing Hb concentration during months 7–9. In total, 186 patients in the efficacy population completed month 9 and 138 completed month 15. Ninety-four patients discontinued the study prematurely (Figure 1) and 49 stopped C.E.R.A. therapy prematurely, most frequently due to patients' request (17/49). Other frequent reasons were the requirement to start dialysis (*n* = 22) and administration of another ESA (*n* = 17) (Figure 1). The mean time between study visits was 35.3 (41.6) days.

The mean age was approximately 51 years, and approximately half the patients were male (Table 1). The mean (SD) eGFR was 35.3 (16.6) mL/min/1.73 m^2^.

Data on immunosuppressive therapy was available for only 43/279 patients (15.4%), including mycophenolic acid (*n* = 24), an mTOR inhibitor (*n* = 18), and a calcineurin inhibitor (*n* = 22).

### 3.2. Iron Status

Iron deficiency, defined as serum ferritin < 100 ng/mL or TSAT < 20%, was present in 26 of the 126 patients for whom data were available at study entry (20.6%). Mean (SD) serum ferritin at study entry was 198 (523) ng/mL (median 72 ng/mL, interquartile range 26–179 ng/mL [*n* = 111]), and mean (SD) TSAT was 28.3 (11.2)% (median 28%, interquartile range 20–35% [*n* = 106]). Use of iron therapy was reported in 74/279 patients in the safety set (26.5%), most frequently ferrous sulfate (*n* = 51) or iron sucrose (*n* = 15).

### 3.3. Previous ESA Therapy and C.E.R.A. Administration

Four-fifths of the population (224/279, 80.3%) were receiving ESA therapy at the time of study entry, most frequently darbepoetin alfa (*n* = 98, 35.1%) or C.E.R.A. (*n* = 45, 16.1%) (Table 1). The cohort of 45 patients previously treated with C.E.R.A. had received the drug for a mean (SD) of 3.7 (3.9) months.

C.E.R.A. was administered subcutaneously in all patients, with three patients also receiving one or more intravenous application (Table 2). The mean (SD) time between C.E.R.A. applications was 34.0 (11.9) days, and the drug was administered in the majority of cases by the patient (Table 2). The mean (SD) dose of C.E.R.A. throughout the study was 95.1 (53.2) µg, with only a small change from the initial dose (92.2 [56.0] *μ*g) to the final dose (98.8 [59.5] *μ*g). No dose changes were required in 119 patients (42.7%). Among the 160 patients (57.3%) in whom the initial C.E.R.A. dose was changed, similar proportions of patients received a dose increase or decrease (Table 2).

### 3.4. Efficacy

**Figure 2:**
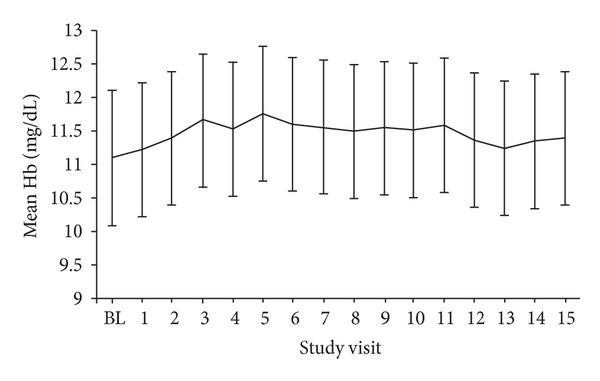
Hb level (efficacy population, *n* = 191). Values are shown as mean (SD). BL: baseline.

At study entry, mean (SD) Hb was 11.1 (0.99) g/dL (median 11.1 g/dL, interquartile range 10.4–11.8 g/dL) in the efficacy population. Mean Hb remained stable throughout the observation period, with values of 11.5 (1.1) g/dL at month 7, 11.6 (1.3) g/dL at month 9, and 11.4 (1.1) g/dL at month 15 (Figure 2). The initial small increase in Hb values during the first three months of the observation period was largely accounted for by initiation of C.E.R.A. in the 55 patients who were ESA-naïve at study entry, in whom mean (SD) Hb increased from 10.8 (0.8) g/dL at baseline to 11.5 (1.0) g/dL at month 3.

No difference in mean baseline Hb values was observed when patients were stratified according to eGFR at study entry. In patients with baseline eGFR < 30 mL/min/1.73 m^2^, mean (SD) Hb was 11.1 (1.2) g/dL (*n*/*N* = 42/193) compared to 11.2 (0.9) g/dL for patients with baseline eGFR 30–60 mL/min/1.73 m^2^.

At study entry, 11.8% (21/178) had an Hb value < 10 g/dL and 3.4% (6/178) had an Hb value > 13 g/dL. At months 7, 9, and 15, respectively, 9.9% (14/142), 10.6% (15/142), and 8.6% (9/105) had an Hb level < 10 g/dL, while 12.7% (18/142), 13.4% (19/142), and 7.6% (8/105) had an Hb level > 13 g/dL. At all times points during the study, no more than 15% of patients had an Hb level ≥ 13 g/dL. During the prespecified evaluation period (visits 7–9), 20.7% of patients (40/193) had all Hb values within the range 11-12 g/dL, increasing to 64.8% for the wider range of 10–13 g/dL (Table 3). As would be expected, the proportion of patients with all Hb within target ranges declined as the period was extended to months 7–12 and 7–15 (Table 3). The mean (SD) deviation in Hb values from the intraindividual mean was 0.50 (0.6) g/dL during the evaluation period (visit 7–9), 1.0 (0.6) g/dL for the period visits 7–12, and 1.2 (0.6) g/dL for the period visits 7–15. During the evaluation period, the majority of patients (87.0%) showed a mean deviation of ≤ 1 g/dL in Hb values from the intraindividual mean (Table 4).

### 3.5. Safety

In total, 55 patients (19.7%) reported a total of 178 adverse events during the study. These included headache in two patients (0.7%) and hypertension in three patients (1.1%) (see Supplementary Table 1 in Supplementary Material available online at http://dx.doi.org/10.1155/2014/179705). Ten adverse events in seven patients (2.5%) were considered by the investigator to be possibly, probably, or definitely related to C.E.R.A. These were hemolytic anemia, pancytopenia, thrombocytopenia, angina pectoris, unstable angina, deep vein thrombosis, hypertension (three patients), and injection site pain. Serious adverse events were reported in 32 patients (11.5%), with four out of 59 events having at least a possible relation with C.E.R.A. (angina pectoris, unstable angina, deep vein thrombosis, and hypertension in one patient each). C.E.R.A. treatment was discontinued in three patients due to adverse events (hypertension; bone marrow depression; pancytopenia with hemolytic anemia) and in four patients due to serious adverse events (dialysis; sepsis with pneumonia, hemodialysis and renal failure; hypertension with angina pectoris; decreased hemoglobin with increased CRP).

There were four deaths during the study, none of which had a suspected relation with C.E.R.A. administration.

Mean (SD) eGFR remained unchanged during the study (study entry, 35.3 [16.6] mL/min/1.73 m^2^; month 15, 34.4 [19.8] mL/min/1.73 m^2^). No consistent pattern of change in serum ferritin concentration or TSAT was observed over the study period. Abnormal erythrocyte counts, as identified by the physician as a clinical deviation from the normal, were reported in 46.7% of patients at the prestudy visit, 24.4% at visit 9, and 23.8% at visit 15. No difference in the rates of clinically significant abnormalities for leukocyte or thrombocyte counts was observed during the study versus prestudy. Other laboratory values including CRP, vitamin B_12_, and liver enzymes showed no clinically relevant changes during the study. Mean blood pressure remained unchanged from baseline (132/77 mmHg) to month 15 (130/77 mmHg).

## 4. Discussion

In this observational study of maintenance kidney transplant patients with stable graft function, C.E.R.A. administered once a month according to local practice achieved a high degree of Hb stability. The main efficacy variable, Hb concentration of 11-12 g/dL at each of the visits at months 7, 8, and 9, was achieved by 20.7% of patients. During the evaluation period, the intrapatient Hb level varied by no more than 1 g/dL in 87% of patients. Hb stability was achieved with a mean time between C.E.R.A. applications of 34 days and with patients self-administering at least some injections in 90% of cases. Moreover, 43% of patients required no change in C.E.R.A. dose throughout the study.

After a small early increase in mean Hb accounted for by C.E.R.A. initiation in the subgroup of patients who were ESA-naïve at study entry, mean Hb remained stable throughout the 15-month observation period. The finding that one in five patients maintained an Hb concentration in the range 11-12 g/dL at months 7, 8, and 9 was consistent with the results of observational studies of C.E.R.A. therapy in patients receiving hemodialysis [37] or peritoneal dialysis [38]. These have reported Hb levels within the 11-12 g/dL window at all three evaluation visits in 15.6% and 18.4% of patients, respectively. These findings should be interpreted against the background of a naturally high degree of Hb variability in patients with CKD [39, 40]. Indeed, it has been shown that the mean within-patient variability is greater than 1 g/dL in CKD patients receiving ESA therapy [40, 41]. Comparisons of Hb stability between the current results and randomized trials of ESA therapies are not clinically relevant since this observational study applied no exclusion criteria for Hb cycling prior to inclusion, in contrast to controlled trials which have typically excluded patients with Hb fluctuation > 1 g/dL during screening [34, 42–44].

One previous study, AnemiaTrans, has examined the use of C.E.R.A. in kidney transplant recipients [45]. AnemiaTrans was a retrospective, multicenter study which included both de novo patients (*n* = 32) and maintenance patients (*n* = 286). As in the current study, the majority of maintenance patients were converted from another ESA therapy to C.E.R.A. Hb levels were monitored for six months from the time of conversion, and consistent with our results, the proportion of patients within the target Hb range of 11–13 g/dL was similar at baseline and at month 6. In the majority of converted patients (52.5%), Hb level fluctuated by less than 1 g/dL between baseline and month 6. The mean C.E.R.A. dose at month 6 (93 *μ*g) was remarkably similar to that used in our population (95.1 *μ*g). The findings of AnemiaTrans, although retrospective, support those of the present study.

The Kidney Disease: Improving Global Outcomes (KDIGO) guidelines for the care of kidney transplant recipients recommend that anemia in kidney transplant patients should be monitored and treated in the same way as patients with CKD [46]. Regular monitoring of Hb levels is mandatory for all recipients [46], but certain subpopulations are at particular risk of anemia. As in the nontransplant population, poor renal function is the strongest predictor [1, 4, 6], but low iron stores [1, 4, 6], probably female gender [4, 6, 47], increasing recipient age [4, 6, 33], donor age [1], poor nutrition, and chronic inflammation [48] also appear to contribute, exacerbated by frequent use of renin-angiotensin-aldosterone system inhibitors [3, 46]. The risk of anemia following transplantation is compounded by immunosuppression with mTOR inhibitors [46, 49–51] or mycophenolic acid [46, 52, 53], although this effect is less marked in the presence of higher GFR [54]. Modification of the immunosuppressive regimen to ameliorate anemia should be considered but may be difficult [46], so management focuses on ESA and iron therapy after exclusion of other causes. A more cautious approach to excess ESA dosing has been adopted since randomized trials in CKD populations indicated an increased risk of stroke and venous thromboembolism when ESA therapy is used to target high Hb levels [22–24], especially in relatively unresponsive patients [55, 56]. In kidney transplantation, a large retrospective study has demonstrated that reaching an Hb level of 14.0 g/dL during ESA therapy is associated with increased mortality compared to 12.5 g/dL [21]. In the current study, fewer than 15% of patients had an Hb level > 13 g/dL at any time point during C.E.R.A. administration.

The recent KDIGO Clinical Practice Guideline for Anemia in Chronic Kidney Disease advises that iron deficiency should be addressed prior to initiation of ESA therapy [57]. In our cohort of patients, the documented use of iron supplementation was low (21.5%), but unfortunately medication reporting and the assessment of iron status seem unlikely to have been comprehensive or fell outside the prespecified windows for study visits. For example, immunosuppressive agents were listed by investigators in only 15% of patients, another clear limitation of the study. Serum ferritin levels, however, indicated the presence of low iron stores in many patients with available data, with median values consistently below the lower recommended limit of 100 ng/mL [32]. Additionally, approximately 25% of patients were below the recommended minimum TSAT level of 20% [32], a level frequently considered to represent functional iron deficiency. While data are incomplete, it appears that iron indices are not routinely monitored or managed at all centers. Thus, our observational study has identified some marked areas of concern where there is room for improvement in patient management, upon which future studies should focus.

Adverse events and serious events judged by the investigator to have at least a possible relation to C.E.R.A. were reported in 2.5% and 1.4% of patients, respectively. Taking into account the comorbidities and multiple concomitant medications given to kidney transplant patients, it is difficult to accurately assign causality to a specific drug. Of the expected adverse events listed in the summary of product characteristics for C.E.R.A., only headache (0.70%) and hypertension (1.10%) were observed, with hypertension contributing to discontinuation in two cases. There were no hematological or biochemical concerns.

An observational study design was chosen to document “real-world” outcomes when patients were selected for C.E.R.A. therapy and managed according to local center practice at a large number of transplant centers. Randomized trials in dialysis-dependent and nondialysis CKD populations have previously shown Hb control to be similar with once-month C.E.R.A. versus more frequent dosing with epoetin or darbepoetin [43, 58, 59], therefore a control arm was not included. It is important to point out that it was neither the aim of this study to demonstrate the efficacy of C.E.R.A., which is already well documented, nor was the goal to compare efficacy between different ESA therapies. The main objective was to gather observational data on Hb fluctuation and C.E.R.A. use in transplanted patients in a real-life setting, which could be used for the development of future interventional trials in this population. Our observational study results provide a basis for future interventional trials of ESA therapy in this population. Given the presence of inadequate iron stores in a substantial proportion of patients future observational studies could benefit from a protocol-stipulated iron supplementation.

## 5. Conclusion

This observational study provides an insight into the use of C.E.R.A. therapy to treat anemia under real-life conditions in a population of stable kidney transplant patients with minimal selection criteria. Once-monthly administration, largely self-administered, achieved stable Hb levels with few dose medications and good tolerability. A once-monthly regimen for ESA therapy may be particularly attractive to transplant recipients who no longer have to attend frequent hemodialysis sessions and are keen to return to a normal lifestyle.

## Supplementary Material

The Supplementary material provides a description of the adverse events reported during the study.

## Figures and Tables

**Table 1:** Patient demographics and baseline characteristics at study entry (safety population, *n* = 279).

Recipient age (years)	51.1 (14.1)
Male recipient, *n* (%)	137 (49.1)
Recipient body mass index (kg/m^2^)	24.8 (4.2)
Cause of end-stage renal disease^a^, *n* (%)	
Glomerulonephritis	92 (33.0)
Polycystic kidney	26 (9.3)
Chronic pyelonephritis	25 (9.0)
Hypertensive nephrosclerosis	19 (6.8)
Diabetic nephropathy	15 (5.4)
Other	96 (34.4)
Unknown	18 (6.5)
Donor age (years)	49.3 (14.4)
Living donor, *n* (%)	54 (19.4)
Time since kidney transplantation (years)	7.2 (6.1)
Hb (g/dL)	11.2 (1.2) g/dL
Iron supplementation, *n* (%)^b^	
Any	74 (26.5)
Intravenous iron	23 (8.2)
Oral iron	56 (20.1)
Unspecified	2 (0.7)
Concomitant medication, *n* (%)	
Mycophenolic acid	24 (8.6)^c^
Calcineurin inhibitor	22 (7.9)^c^
mTOR inhibitor	18 (6.5)^c^
Angiotensin-converting enzyme inhibitor	107 (38.4)
Angiotensin-II receptor antagonist	95 (34.1)
eGFR (MDRD) at study entry, mL/min/1.73 m^2^	
Mean (SD)	35.3 (16.6)
Median (interquartile range)	33.5 (24.0–44.0)
Serum ferritin, ng/mL	
Mean (SD)	198 (523)
Median (interquartile range)	72 (26–179)
Transferrin saturation, %	
Mean (SD)	28.3 (11.2)
Median (interquartile range)	28 (20–35)
CRP, mg/L	
Mean (SD)	8.4 (21.0)
Median (interquartile range)	3.0 (1.2–6.4)
Previous ESA therapy, *n* (%)	
None	55 (19.7)
Darbepoetin alfa	98 (35.1)
C.E.R.A.	45 (16.1)
Epoetin beta	61 (21.9)
Epoetin delta	13 (4.7)
Epoetin alfa	7 (2.5)
Duration of previous ESA therapy, months	
Darbepoetin alfa	20.2 (22.3)
C.E.R.A.	3.7 (3.9)
Epoetin beta	19.6 (18.3)
Epoetin delta	16.5 (18.7)
Epoetin alfa	12.8 (9.4)

^a^More than one cause could be listed per patient. ^b^More than one type could be listed per patient. ^c^Data on immunosuppressive therapy were provided in only 43 patients.

Continuous variables are shown as mean (SD) unless otherwise stated.

C.E.R.A.: continuous erythropoietin receptor activator; CRP: C-reactive protein; eGFR: estimated GFR; ESA: erythropoiesis stimulating agent; MDRD: Modification of Diet in Renal Disease; SD: standard deviation.

**Table 2:** C.E.R.A. administration (safety population, *n* = 279).

Reason for initiation of C.E.R.A., *n* (%)^a^	
New and innovative application scheme	173 (62.2)
No previous ESA therapy	86 (30.9)
Therapeutic failure of previously used ESA	16 (5.8)
Adverse effects of previously used ESA	2 (0.7)
Other	5 (1.8)
Route of application, *n* (%)	
Subcutaneous	260 (93.2)
Subcutaneous and intravenous	3 (1.1)
Unknown	16 (5.7)
Application by, *n* (%)	
Patient	180 (64.5)
Patient or nurse	1 (0.4)
Physician	28 (10.0)
Physician or patient	69 (24.7)
Physician or patient or nurse	1 (0.4)
C.E.R.A. dose per application, µg	
Initial dose	
Mean (SD)	92.2 (56.0)
Median (range)	75.0 (30–360)
Final dose, mean (SD)	
Mean (SD)	98.8 (59.5)
Median (range)	75.0 (30–360)
Throughout study	
Mean (SD)	95.1 (53.2)
Median (range)	76.9 (30–360)
Time between C.E.R.A. applications, days	
Mean (SD)	34.0 (11.9)
Median (range)	31.2 (13–91)
C.E.R.A. dose changes, *n* (%)	
No dose change	119 (42.7)
Any dose change	160 (57.3)
Any dose decrease	117 (41.9)
Any dose increase	132 (47.3)

C.E.R.A.: continuous erythropoietin receptor activator; SD: standard deviation.

^a^More than one reason could be selected from a preprinted list.

**Table 3:** Proportion of patients within Hb target ranges (efficacy population).

Hb range	Visit window		
	7–9^a^	7–12	7–15
11-12 g/dL	20.7% (40/193)	2.9% (4/137)	0.0% (0/153)
11–13 g/dL	40.4% (78/193)	21.2% (29/137)	15.0% (23/153)
10–12 g/dL	42.0% (81/193)	24.1% (33/137)	14.4% (22/153)
10–13 g/dL	64.8% (125/193)	52.6% (72/137)	43.1% (66/153)

^a^Prespecified evaluation period.

**Table 4:** Deviation of Hb from intraindividual mean values.

Deviation	Visit window		
	7–9^a^	7–12	7–15
≤1 g/dL	87.0% (168/193)	57.7% (79/137)	41.8% (64/153)
>1 to 2 g/dL	9.3% (18/193)	32.8% (45/137)	45.8% (70/153)
>2 g/dL	3.6% (7/193)	9.5% (13/137)	12.4% (19/153)

^a^Prespecified evaluation period.

Calculations are based on maximum deviation from individual mean values.
