# Supplementary material for: Anemia Control in Kidney Transplant Recipients Using Once-Monthly Continuous Erythropoietin Receptor Activator: A Prospective, Observational Study
Source: J Transplant. 2014 May 4;2014:179705. doi: 10.1155/2014/179705 (PMC4026977; doi:10.1155/2014/179705)
Supplement: Supplementary file 1 — The Supplementary material provides a description of the adverse events reported during the study. [file 179705.f1.doc]

**Supplementary Table 1**. Adverse events, n (%) (N=279)

| Any adverse event | 55 (19.7) |
| --- | --- |
| Diarrhea | 10 (3.6) |
| Nasopharyngitis | 9 (3.2) |
| Urinary tract infection | 9 (3.2) |
| Pneumonia | 5 (1.8) |
| Blood creatinine increased | 4 (1.4) |
| Cough | 4 (1.4) |
| Influenza like illness | 4 (1.4) |
| Nausea | 4 (1.4) |
| Gastroenteritis | 3 (1.1) |
| Hemoglobin decreased | 3 (1.1) |
| Hypertension | 3 (1.1) |
| Oral herpes | 3 (1.1) |
| Abdominal pain upper | 2 (0.7) |
| Arthralgia | 2 (0.7) |
| Dyspepsia | 2 (0.7) |
| Fatigue | 2 (0.7) |
| Headache | 2 (0.7) |
| Hypoesthesia | 2 (0.7) |
| Musculoskeletal discomfort | 2 (0.7) |
| Upper respiratory tract infection | 2 (0.7) |
| Weight decreased | 2 (0.7) |
| Abdominal hernia | 1 (0.4) |
| Alopecia | 1 (0.4) |
| Anemia | 1 (0.4) |
| Angina pectoris | 1 (0.4) |
| Angina unstable | 1 (0.4) |
| Angioedema | 1 (0.4) |
| Aphthous stomatitis | 1 (0.4) |
| Back pain | 1 (0.4) |
| Basal cell carcinoma | 1 (0.4) |
| Bone marrow failure | 1 (0.4) |
| Bone pain | 1 (0.4) |
| Bronchitis | 1 (0.4) |
| Carpal tunnel decompression | 1 (0.4) |
| Cerebral infarction | 1 (0.4) |
| Chest pain | 1 (0.4) |
| Contusion | 1 (0.4) |
| Coronary artery disease | 1 (0.4) |
| C-reactive protein increased | 1 (0.4) |
| Cytomegalovirus infection | 1 (0.4) |
| Deep vein thrombosis | 1 (0.4) |
| Depression | 1 (0.4) |
| Device failure | 1 (0.4) |
| Dialysis | 1 (0.4) |
| Diplopia | 1 (0.4) |
| Dyspnea exertional | 1 (0.4) |
| Dysuria | 1 (0.4) |
| Eczema | 1 (0.4) |
| Edema peripheral | 1 (0.4) |
| Epicondylitis | 1 (0.4) |
| Gait disturbance | 1 (0.4) |
| Gastroenteritis norovirus | 1 (0.4) |
| Groin pain | 1 (0.4) |
| Hemodialysis | 1 (0.4) |
| Hemolytic anemia | 1 (0.4) |
| Herpes simplex | 1 (0.4) |
| Herpes zoster | 1 (0.4) |
| Hirsutism | 1 (0.4) |
| Human polyomavirus infection | 1 (0.4) |
| Hyperhidrosis | 1 (0.4) |
| Hypocalcemia | 1 (0.4) |
| Inflammation | 1 (0.4) |
| Intervertebral discitis | 1 (0.4) |
| Kidney transplant rejection | 1 (0.4) |
| Leukocytosis | 1 (0.4) |
| Ligament rupture | 1 (0.4) |
| Ligament sprain | 1 (0.4) |
| Liver function test abnormal | 1 (0.4) |
| Localized infection | 1 (0.4) |
| Macrocytosis | 1 (0.4) |
| Malaise | 1 (0.4) |
| Molluscum contagiosum | 1 (0.4) |
| Multi-organ failure | 1 (0.4) |
| Neck pain | 1 (0.4) |
| Neoplasm skin | 1 (0.4) |
| Nephrotic syndrome | 1 (0.4) |
| Night sweats | 1 (0.4) |
| Otitis media | 1 (0.4) |
| Pain in extremity | 1 (0.4) |
| Palpitations | 1 (0.4) |
| Pancytopenia | 1 (0.4) |
| Papilloma | 1 (0.4) |
| Parathyroidectomy | 1 (0.4) |
| Phantom pain | 1 (0.4) |
| Productive cough | 1 (0.4) |
| Psychomotor hyperactivity | 1 (0.4) |
| Pyrexia | 1 (0.4) |
| Renal cancer | 1 (0.4) |
| Renal failure | 1 (0.4) |
| Renal failure acute | 1 (0.4) |
| Sepsis | 1 (0.4) |
| Septic shock | 1 (0.4) |
| Shunt occlusion | 1 (0.4) |
| Sinusitis | 1 (0.4) |
| Sudden cardiac death | 1 (0.4) |
| Thrombocytopenia | 1 (0.4) |
| Tinnitus | 1 (0.4) |
| Transient ischemic attack | 1 (0.4) |
| Tremor | 1 (0.4) |
| Tympanic membrane disorder | 1 (0.4) |
| Urinary tract infection enterococcal | 1 (0.4) |
| Urosepsis | 1 (0.4) |
| Varicella | 1 (0.4) |
| Vertigo | 1 (0.4) |
| VIth nerve disorder | 1 (0.4) |
| Vomiting | 1 (0.4) |
| Walking disability | 1 (0.4) |
| Wound infection | 1 (0.4) |
